# P167: The challenges of implementing patient participation in hand hygiene – results of a qualitative inquiry in the framework of a randomized controlled effectiveness trial

**DOI:** 10.1186/2047-2994-2-S1-P167

**Published:** 2013-06-20

**Authors:** S Touveneau, L Clack, F Da Liberdade Jantarada, A Stewardson, M Schindler, M Bourrier, D Pittet, H Sax

**Affiliations:** 1 Infection Control Program, University of Geneva Hospitals, Geneva 14, Switzerland; 2 Division of Infectious Diseases and Hospital Epidemiology, University Hospital Zurich, Zurich, Switzerland; 3 Departement of Sociology, University of Geneva, Geneva, Switzerland

## Introduction

A single center, cluster randomized controlled effectiveness trial was conducted to compare two novel hand hygiene (HH) promotional strategies. Sixty six wards were randomized to standard HH promotion (control), standard promotion plus HH performance feedback (FB), or FB plus active patient participation (PP). While FB seemed to be well accepted by HCWs, the introduction of PP proved to be more challenging.

## Objectives

Here we focus on PP and report the results of a qualitative inquiry, designed to investigate the range of success with its implementation.

## Methods

A ward-level case study was conducted through 3 focus groups with ward staff and 6 interviews with ward head nurses. Participants were selected following an extremes sampling strategy regarding adoption strength. The hospital’s infection control nursing staff participated in additional focus group. All sessions were audiotaped and transcribed verbatim. Analysis was deductive (using a framework of themes that had been previously established) and inductive (grounded theory).

## Results

Four main and 12 sub-themes (in parenthesis) emerged: Context (patient emancipation, behavioral norms, experiential stages of change, infrastructure); Risk knowledge (nil); Interaction (means of communication, emotions); Implementation process (study and implementation design, message delivery, leadership, unintended effects, time and workload, sustainability). The action of confronting another person about a failure became less emotionally menacing through positive experiences, innovative means of communication, and institutionalization of the project. Individual leadership engagement had a major impact on implementation success. Exclusion from intervention arms motivated control wards to improve HH performance independently.

## Conclusion

This qualitative research permitted to explain the quantitative study findings and to explore the evolutionary and complex implementation process of PP. While PP remains challenging, it is our hope that the findings of this study may facilitate future patient participation projects.

## Disclosure of interest

None declared

